# Artificial intelligence in genitourinary pathology

**DOI:** 10.1111/his.70020

**Published:** 2025-12-12

**Authors:** Ankush U Patel, Anil V Parwani, Swati Satturwar

**Affiliations:** ^1^ The Ohio State University, Wexner Medical Center and James Cancer Center Columbus OH USA

**Keywords:** artificial intelligence, bladder cancer, computational pathology, diagnostic orchestrator, digital transformation, foundation models, genitourinary pathology, Gleason grading, ORCHESTRATE framework, prostate cancer, renal cancer, ROI pathology, VALIDATED framework

## Abstract

Artificial intelligence (AI) is now a practical, value‐generating tool in genitourinary (GU) pathology. Real‐world deployments report up to 65% time‐savings and multi‐million‐dollar returns on investment within 3 years at high‐volume centres. Across prostate, bladder, renal and testicular systems, contemporary algorithms equal or exceed expert accuracy for cancer detection, grading and prognostication. Foundation models trained on millions of whole‐slide images now match specialized organ‐specific tools without bespoke tuning. High AI–pathologist concordance is widely regarded as a surrogate marker of safety and clinical acceptability, yet no universally codified regulatory threshold for sensitivity, specificity or concordance has been issued. Because internationally recognized guidelines still omit detailed instructions for safe roll‐out and sustained performance, we distilled insights from real‐world deployments and pioneering pilot studies into two complementary roadmaps: the nine‐step VALIDATED framework, which focuses on governance and safety oversight, and the 11‐principle ORCHESTRATE blueprint, which guides day‐to‐day implementation. By 2030, we anticipate AI will automate ~80% of routine quantification, allowing pathologists to assume the role of diagnostic orchestrators who integrate multimodal data streams, helping offset a ~40% workforce shortfall and reducing inter‐observer variability across practice settings. This review distils the evidence, economics and practical guidance required for successful AI adoption in GU pathology. Institutions following the VALIDATED–ORCHESTRATE pathway can harness efficiency gains while maintaining diagnostic excellence and achieving positive ROI within 5 years.

## Graphical abstract

Artificial intelligence (AI) is now practical in genitourinary pathology. We synthesize evidence and economics into a two‐part playbook: VALIDATED (governance) and ORCHESTRATE (operations), to safely deploy AI tools across prostate, bladder, kidney and testis. This pathway delivers 20%–65% time savings and positive ROI, enabling pathologists as ‘diagnostic orchestrators’ by 2030.

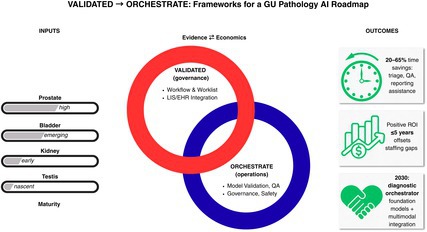

**Abbreviations**
AI: artificial intelligence
AUC: area under the curve
BCG: bacillus Calmette–Guérin
GCNIS: germ cell neoplasia in situ
GU: genitourinary
HIFs: human‐interpretable features
ICC: immunocytochemistry
KPI: key performance indicator
LVI: lymphovascular invasion
OSU: Ohio State University
RCC: renal cell carcinoma
ROI: region of interest
TATs: turnaround times
TGCT: testicular germ cell tumour
TIL: tumour‐infiltrating lymphocyte
WSI: whole‐slide images

## Introduction

Artificial intelligence has crossed the threshold from experimental technology to operational tool in pathology.^1^, ^2^, ^3^, ^4^, ^5^, ^6^ Genitourinary pathology presents unique opportunities for AI adoption: rich morphological complexity, nuanced diagnostic challenges, increasing cancer burden, complex diagnostic inputs spanning imaging to molecular data and mature computational infrastructure capable of handling gigapixel whole‐slide images (WSI).^3^, ^4^, ^7^, ^8^, ^9^, ^10^, ^11^, ^12^, ^13^, ^14^ Prostate, bladder and renal cancers have proven fertile ground for AI tools that excel at cancer detection, tumour subtyping, grading and prognostication.^10^, ^12^ Real‐world deployments demonstrate meaningful efficiency gains, with reduced turnaround times (TATs), decreased ancillary immunohistochemical testing and optimized laboratory resource utilization.^15^, ^16^, ^17^, ^18^, ^19^

Regulatory bodies have begun approving AI tools for specific applications like cancer detection, yet comprehensive frameworks for fully autonomous diagnostic AI remain underdeveloped.^2^, ^5^, ^16^, ^20^, ^21^, ^22^, ^23^ The current landscape represents transitional uncertainty, highlighting the need for clear guidance and robust validation strategies.^15^, ^16^, ^17^, ^18^, ^19^

Practical lessons and strategic insights into future advancement emerge from examining successes and limitations of current AI solutions across GU systems. This transitional period to 2030 offers valuable opportunities. Gaps in existing approaches provide beacons guiding the development of more sophisticated, reliable and clinically valuable AI solutions for GU pathology.

### Conventions Used in this Review

Unless otherwise stated, percentage improvements (e.g. time savings, negative slide exclusion) are relative to digitized whole‐slide imaging workflows without AI assistance (standard laboratory information systems [LIS]/VNA integration, routine QC). Where a study's comparator is an analogue glass workflow, we state this explicitly.

## Prostate Pathology: High Volume, High Stakes and the Illusion of Perfection

The advancement of AI for prostate pathology is attributable to domain‐specific catalysts including high biopsy volumes, pattern‐based grading systems, and extensive annotated data sets including PANDA, CAMELYON‐PCam and STHLM3.^24^, ^25^, ^26^, ^27^, ^28^, ^29^, ^30^, ^31^ The PANDA challenge, with 10,616 WSIs across different scanner platforms and staining protocols, established a blueprint for developing AI tools that perform consistently across real‐world clinical settings.^24^, ^32^ Prostate cancer represents approximately 40% of genitourinary histopathology workload in Western laboratories, further incentivizing AI adoption.^32^, ^33^ Deep learning models now demonstrate exceptional performance in differentiating benign from malignant tissues, routinely achieving area under the curve (AUC) scores exceeding 0.98, with multi‐centre validation studies reporting ≥0.99.

**Figure 1:**
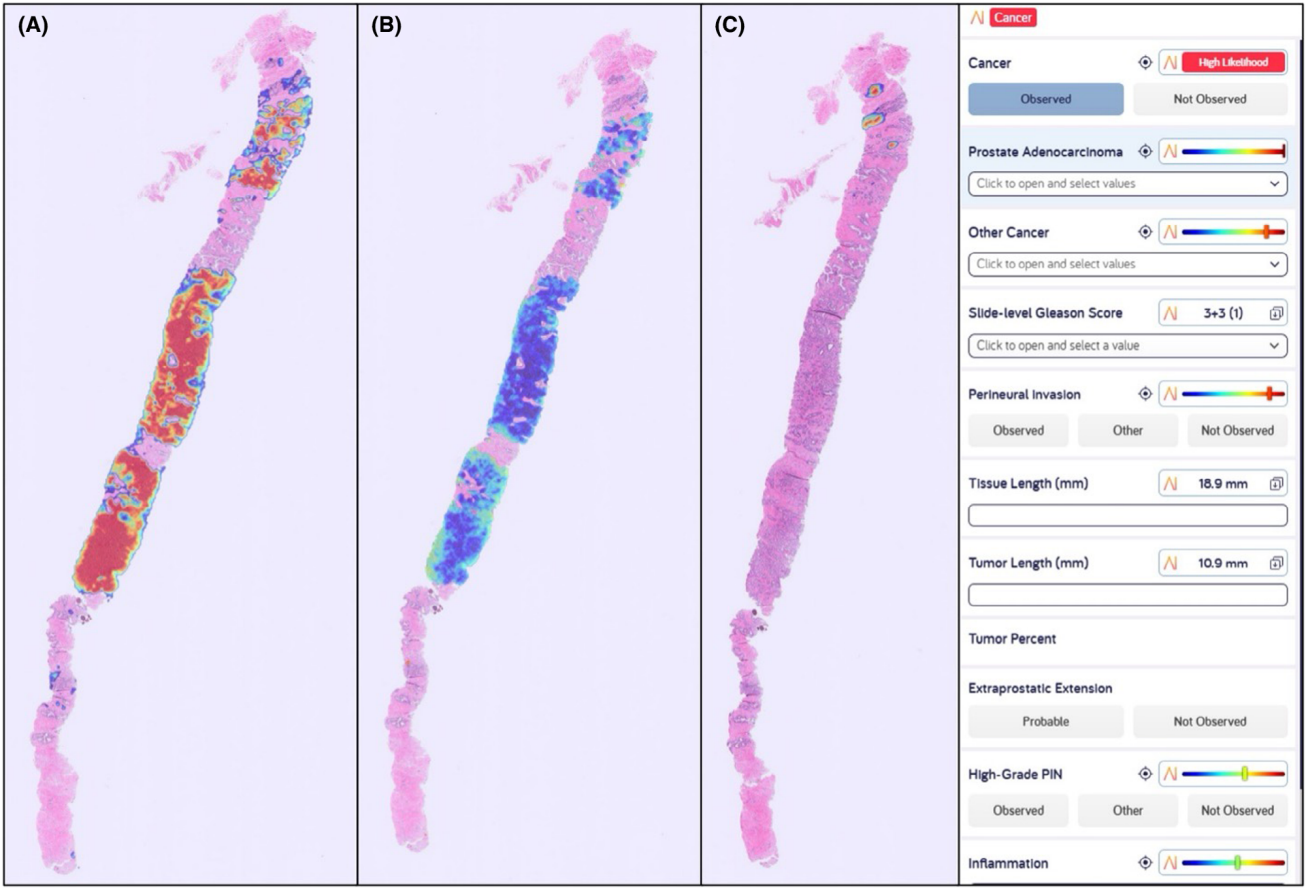
Prostate‐biopsy heat‐map generated with Galen™ Prostate (Ibex Medical Analytics, Tel Aviv, Israel; CE‐IVD marked). (**A**) Red heatmap highlights cancer foci, (**B**) blue heatmap showing colour‐coded Gleason grade (3 + 3), (**C**) red–yellow heatmap showing perineural invasion foci.

FDA approval of Paige Prostate Detect in 2021 for prostate cancer detection in core needle biopsies marked the dawn of commercial AI adoption in pathology across US centres.^24^, ^34^, ^35^, ^36^ CE‐marked solutions including Ibex's Galen Prostate (Figure 1), Aiforia's Prostate Cancer AI and DeepDx are gaining traction in European laboratories (Table 1).^37^, ^38^, ^39^, ^40^, ^41^, ^42^

**Table 1:** Commercially available AI tools for prostate cancer diagnosis and screening

Product	Company	Detection	Grading	Quantification	Approvals
Paige Prostate Detect/Grade and Quantify	Paige AI (USA)	✓	✓	✓	FDA (Detect); CEIVD
Galen Prostate	Ibex (Israel)	✓	✓	✓	CEIVD
Aiforia Prostate	Aiforia (Finland)	✓	✓	✓	CEIVD
DeepDx	Deep Bio (South Korea)	✓	✓	✓	CEIVD, MFDS
Inify Prostate	Inify Laboratories (Sweden)	✓	✓	–	CEIVD
HALO Prostate	Indica Labs (USA)	✓	✓	–	CEIVD

Adoption is progressing in validated frameworks such as Finland's Fimlab Laboratories, France's Medipath network, and the multinational EMPAIA consortium.^40^, ^43^ Reports from The Ohio State University (OSU) Wexner Medical Center indicate AI‐assisted prostate workflows save 20%–25% of pathologists' time by automating manual tasks and diagnostic data synthesis (automated aggregation, integration and presentation of multiple diagnostic parameters extracted from WSIs including collation of quantitative features like tumour area, grade group, glandular architecture, Gleason pattern quantitation, tumour burden, nuclear and glandular morphometry, perineural invasion, extraprostatic extension and presence of histologic variants or secondary patterns).^44^ Cases with unusual findings can be flagged to optimize review order and reduce TAT. Outside OSU, studies using Paige Prostate and other CE‐marked AI solutions report substantial efficiency gains of up to 65% in AI‐assisted pre‐screening or concurrent reads.^45^ A Swedish microsimulation projected an 80% reduction in biopsy cores requiring manual review without compromise to detection of clinically significant cancers.^46^, ^47^, ^48^, ^49^

Results from deployments and prospective studies must be weighed with careful interpretation of study results. Despite being signified by milestone outcomes (e.g. quadratically weighted Cohen's *κ* values of approximately 0.862–0.868, indicating strong expert concordance), 13%–14% of cases included in the PANDA challenge resulted in clinically meaningful disagreements.^24^, ^27^, ^42^, ^50^, ^51^ Sensitivity rates (97%–98%) combined with lower specificity (75%–84%) suggest a propensity for AI to flag benign tissues as suspicious, aligning with the current dominance of high‐sensitivity ‘find‐the‐needle’ tasks like negative‐case exclusion, case triage or region‐of‐interest highlighting, in commercially deployed AI applications for anatomic pathology. These results support AI's utility as a preliminary screening tool to identify cases for human review rather than as absolute diagnostic instruments.^52^, ^53^, ^54^, ^55^, ^56^

AI limitations in prostate pathology still manifest in edge cases: atypical glandular proliferation, staining variability and identification of rare subtypes of prostate adenocarcinoma like foamy gland or pseudohyperplastic adenocarcinoma.^57^ Models trained predominantly on conventional adenocarcinoma with common patterns may misclassify rare subtypes or exhibit false confidence when faced with artefacts resembling tumour morphology.^58^ Biases present in training data sets, often due to interobserver variability among pathologists, may inadvertently be amplified by AI, compromising diagnostic consistency.^42^ External validation studies show significant performance drops when AI models are applied across varying institutions or scanning technologies, illustrating challenges in achieving true generalizability.^45^, ^59^, ^60^, ^61^, ^62^, ^63^, ^64^, ^65^ Clinically, AI integration introduces new operational considerations including trust in AI‐driven screening, liability concerns related to missed diagnoses, reimbursement and documentation standards for AI‐assisted evaluations. Emerging decision points invoke the need for clear guidelines and best practices.^24^, ^66^

Prostate pathology remains the most mature example of AI integration within GU pathology and of successful human–AI collaboration. AI‐assisted GU pathology workflows report tangible improvements in efficiency, reduced diagnostic TAT and reduced reliance on ancillary immunohistochemical studies and related expenditures including secondary consultations—lowering associated costs and accelerating case reporting by approximately 20%.^15^ These improvements are supported by maintenance of diagnostic safety and quality.^67^

Prostate AI demonstrates the potential to standardize diagnostic grading, significantly reduce interobserver variability and enhance consensus among pathologists. Quantitative tools for prostate pathology (e.g. precise estimation of Gleason pattern percentages and differentiation of cribriform versus fused patterns) aid in focusing diagnostic interpretation. Prospective validation studies must continuously verify the validity of AI‐detected regions (e.g. to confirm rare or high‐grade lesions are not overlooked).^30^, ^68^, ^69^ The clinical value of prostate‐cancer AI ultimately depends on demonstrable gains in diagnostic TAT, reduced ancillary testing, and more uniform patient stratification—improvements that together translate into earlier treatment decisions and lower system costs.

## Bladder Pathology: Complexity of Cues and the Drive for Consistency

AI research in bladder pathology is advancing quickly, although routine clinical use trails behind prostate applications. Current platforms span digital histopathology, urine cytology and cystoscopy, each aimed at enhancing diagnostic accuracy, workflow efficiency and inter‐observer consistency.

AI algorithms now exceed 80% accuracy for tumour detection, grade prediction and tissue‐component segmentation (urothelium, stroma, muscle).^70^ Beyond histological read‐outs, systems have been trained to forecast recurrence, progression, response to Bacillus Calmette–Guérin (BCG) therapy and the likelihood of cystectomy in high‐risk non‐muscle‐invasive disease.^70^ Lymph‐node metastasis has been reported with AUC 0.978–0.998, enabling exclusion of 80%–92% of true‐negative slides from exhaustive review, aiding pathologist throughput.^71^

**Figure 2:**
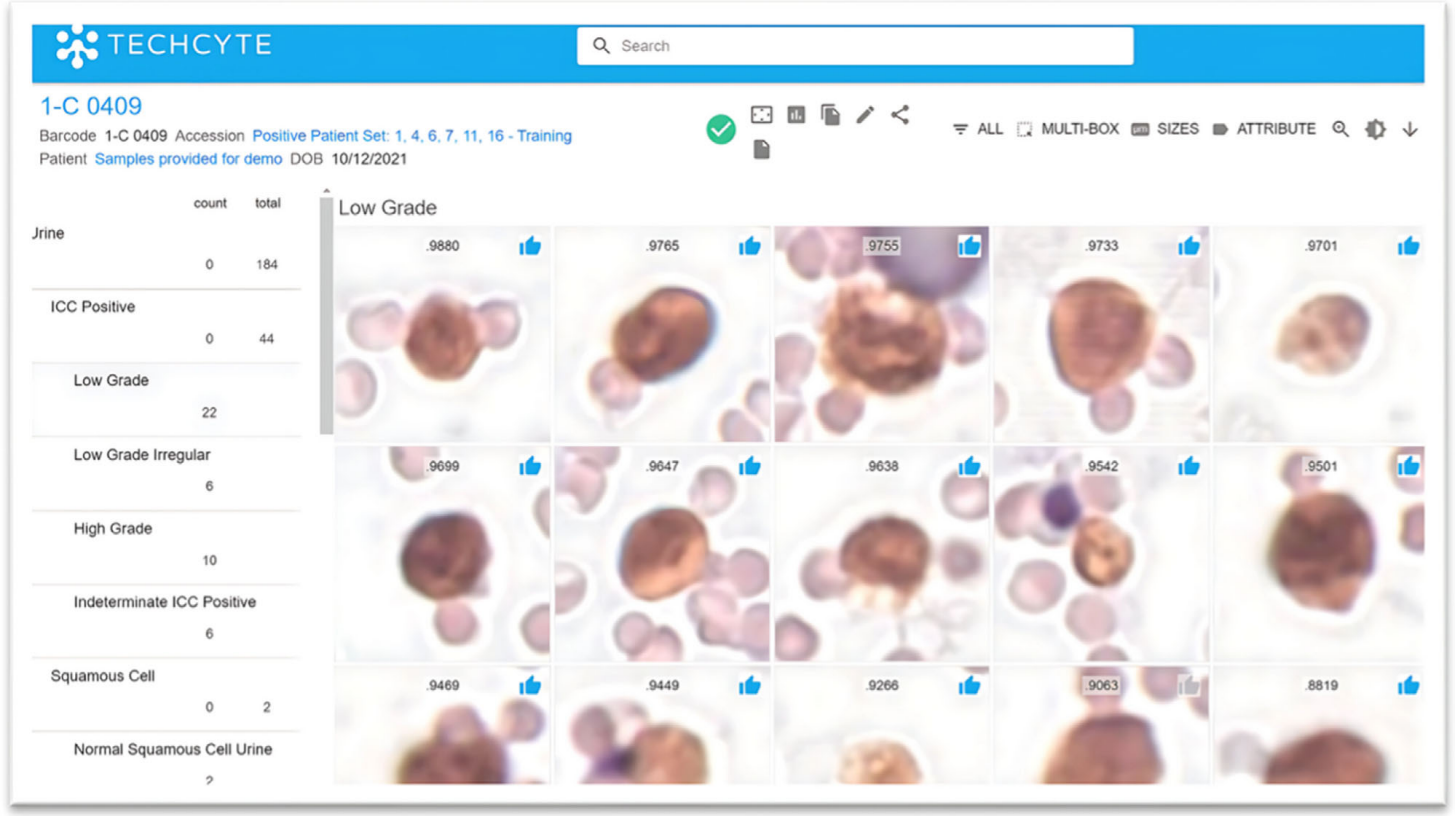
Techcyte/Cytobay bladder cancer screening solution.^82^, ^83^ The Techcyte bladder cancer screening solution analyses urine samples to identify immunocytochemistry‐positive low‐grade, high‐grade and other ‘diagnostically significant’ cells utilizing a proprietary staining technique (CytoBay Inc.).^82^, ^83^

Regulatory traction remains modest. The TOBY test received FDA Breakthrough Device Designation in 2025, with pivotal validation under way.^72^ Other tools, including VisioCyt (VitaDX), Menarini/Nucleix, Techcyte/Cytobay and URO17 (Lumea + AIRA Matrix), are CE‐marked or in late‐stage evaluation (Figure 2).^73^, ^74^, ^75^, ^76^, ^77^, ^78^, ^79^, ^80^, ^81^

Meta‐analyses consistently show AI assistance raising pathologist sensitivity and reducing subjective variability in grading and staging.^84^, ^85^ Limited interpretability, domain‐shift vulnerability and still‐evolving regulatory pathways remain hurdles.^86^, ^87^

Workflow studies illustrate tangible benefits. Lymph‐node algorithms safely exclude 80%–92% of true‐negative slides from exhaustive review, accelerating throughput.^71^ Multimodal prognostic models integrate histologic and molecular features to personalize therapy for muscle‐invasive tumours.^88^, ^89^ Automated grading systems, validated across international cohorts, now approach expert‐level accuracy and reproducibility.^57^, ^90^, ^91^, ^92^, ^93^, ^94^ Large‐scale slide processing demonstrates substantial workload reduction without compromising diagnostic quality.^95^, ^96^, ^97^ Pilot decision‐support systems already feed AI‐derived risk scores into pathologist worklists and tumour‐board discussions.^70^, ^98^, ^99^ In practice, AI diagnostic outputs are only as reliable as the data quality and quantity they receive. In a prospective, 150‐patient urine‐cytology study, a liquid immunocytochemistry (ICC) platform successfully optimized pre‐analytical protocols to ensure adequate cellularity (≥2644 urothelial cells/slide), in turn enabling enhanced downstream AI diagnostic accuracy.^100^

The complex histology of bladder tumours and artefact‐prone nature of transurethral resection specimens present unique obstacles to AI. Crush artefact, cautery distortion and dense inflammation can trigger false‐positive invasion calls.^70^ Long‐standing human inter‐observer variability complicates the creation of consistent training labels, risking systematic bias.^101^, ^102^ Rare variants, including plasmacytoid, micropapillary and others, remain under‐represented in training data sets, limiting algorithmic sensitivity.^93^, ^103^, ^104^, ^105^, ^106^

As nuanced complexities are ameliorated with advances, AI will serve essential roles in clinical workflows for bladder cancer. AI‐driven triage could prioritize high‐risk cases, whereas molecular surrogates (e.g. FGFR3‐mutation prediction) may streamline patient selection for targeted therapies.^106^ In multidisciplinary tumour boards, quantitative AI outputs already enhance communication and decision‐making.^107^, ^108^ Risk‐stratification models for high‐risk non‐muscle‐invasive disease have shown clinical utility across centres and are entering prospective evaluation.^98^, ^109^ Sustained clinician trust will depend on transparent, continuously validated and explainable systems.^31^, ^110^, ^111^, ^112^, ^113^, ^114^

## Kidney Pathology: Emerging Potential Amid Data Challenges

AI research in renal pathology is expanding, with algorithms that segment renal compartments, quantify interstitial fibrosis, tubular atrophy and glomerulosclerosis and classify renal cell carcinoma (RCC) subtypes at AUC > 0.93; clear‐cell RCC grading reaches AUC 0.89–0.96.^115^, ^116^, ^117^, ^118^, ^119^, ^120^, ^121^, ^122^ Clinical translation, however, is slowed by limited data, demanding validation protocols and evolving regulatory requirements.^122^, ^123^, ^124^ Predictive models are beginning to forecast clinical outcomes, such as response to anti‐angiogenic therapy, and to bring quantitative objectivity to immunofluorescence and electron‐microscopy images.^122^, ^125^, ^126^

Progress is constrained by small, single‐centre data sets and the need to integrate multimodal inputs (bright‐field, immunofluorescence, electron microscopy and clinical–radiologic data).^122^, ^124^, ^127^, ^128^, ^129^ Renal disease heterogeneity compounds the stagnancy: subtle or focal lesions demand expert annotation across glomeruli, tubules, interstitium and vessels, yet public repositories (e.g. TCGA) lack detailed histological labels.^130^, ^131^, ^132^, ^133^, ^134^, ^135^ Precise annotation of mixed‐histology specimens, for example, tumour versus non‐neoplastic tissues, or rare prognostically significant variants remains difficult.^136^

Amidst these challenges, deep learning models have distinguished clear cell, papillary and chromophobe RCC with high accuracy.^119^, ^137^, ^138^, ^139^ Automated clear‐cell RCC grading using Fuhrman nuclear grading has reported AUC scores (0.85–0.92).^140^, ^141^ Multi‐institutional studies using TCGA images distinguish tumour, normal and non‐neoplastic tissue despite marked histological diversity (F1 ≈ 0.88, AUC ≈ 0.97).^142^, ^143^, ^144^, ^145^ Early work also infers von Hippel–Lindau‐pathway mutations directly from H&E slides (AUC 0.75–0.85), pointing towards future pathology–genomic integration.^146^

Rare RCC variants and mixed‐histology specimens remain underrepresented, making broad generalization impossible without multi‐centre data sharing.^130^, ^131^, ^132^, ^133^, ^134^, ^135^, ^136^

**Figure 3:**
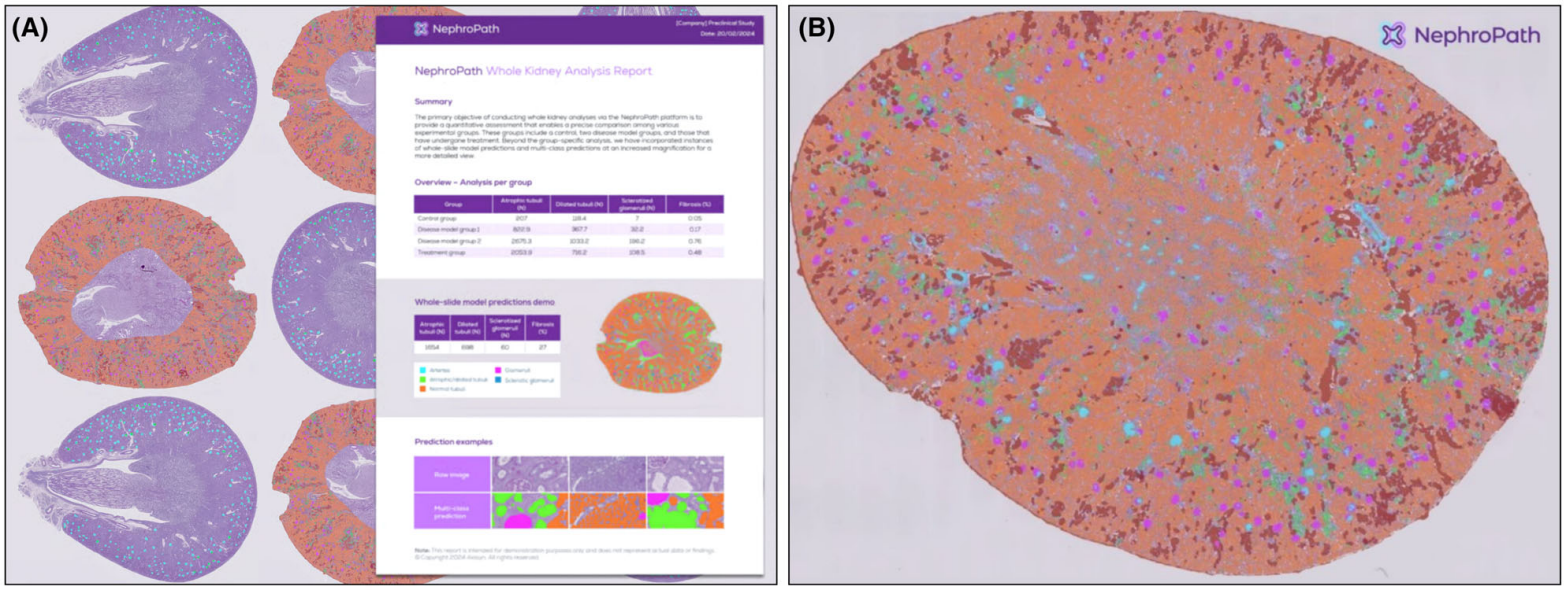
Aiosyn NephroPath. (**A**) NephroPath (research use only) whole kidney analysis report including precise comparison of experimental groups (control, two disease model groups, and treatment groups), whole‐slide model predictions and multi‐class predictions.^147^ (**B**) Quantification of classified structures including atrophic tubuli, dilated tubuli, sclerotized glomeruli, and fibrosis (%).^147^

In the near‐term, AI for renal pathology is poised to function as an adjunct, triaging biopsies, flagging subtle lesions for subspecialist review, quantifying tumour burden and packaging subtype, grade and imaging features into unified risk reports for multidisciplinary teams (Figure 3).

## Testis Pathology: Data Scarcity and the Potential of Transfer Learning

AI development in testicular germ cell tumours (TGCTs) remains at an early, experimental stage, with minimal clinical integration and limited impact on current diagnostic workflows.^148^ Early studies have reported ~89% sensitivity (F1 ≈ 0.88) for tumour‐infiltrating lymphocytes (TIL) detection, feasibility for lymphovascular invasion (LVI) detection with modest precision requiring external validation, and reductions in observer variability.^148^, ^149^, ^150^, ^151^

The near‐term clinical value of AI in testis pathology lies in addressing challenges posed by rare, histologically complex tumours. Initial AI models trained on limited data sets (<200 digitized slides) reported F1 scores ~0.92 for TGCT versus benign classification and subtype true‐positive rates of 75%–95%.^148^ AI could serve as a screening adjunct, reviewing orchiectomy specimens for small tumour foci that might otherwise be overlooked, aiding in detecting subtle LVI and quantifying germ cell neoplasia in situ (GCNIS), standardizing margin and adjacent tissue assessments. Transfer learning, pretraining on high‐volume tissues (e.g. prostate, bladder) and fine‐tuning on TGCT, is a practical strategy to improve performance by leveraging morphological similarities across tumour types, enabling more nuanced differentiation within mixed germ‐cell tumours. Collaborative multi‐institutional data pooling will be essential to overcome dataset limitations and support robust, generalizable AI tools.

Near‐term clinical roles for AI in testis pathology will likely remain supportive rather than definitive, especially in academic and specialized centres. AI could expedite labour‐intensive tasks, such as quantifying tumour composition in mixed TGCTs or assisting intraoperative frozen‐section evaluations. Cloud‐based tools may extend expertise remotely and help reduce diagnostic disparities in resource‐limited settings. Comprehensive, AI‐powered digital pathology atlases can support education and practical second‐opinion consultations.

Advances in foundation models and vision–language systems could enhance small‐data set performance by transfer and text supervision from literature‐derived descriptors (e.g. associating captions like ‘Schiller–Duval bodies’ with histologic patterns). These approaches may help recognize rare entities when visual exemplars are scarce.

Continued progress depends on rigorous validation, multi‐institutional collaboration and careful data set curation, highlighting the central role of pathologists in shaping effective AI solutions in this domain.

### Cross‐Cutting Data Limitations for Low‐Prevalence Entities

The scarcity of comprehensive, well‐labelled data sets increases risks of overfitting and limits generalizability across sites and scanners. Rare variants and mixed‐component tumours (e.g. mixed TGCTs) are under‐represented, making class boundaries unstable. Inconsistent archival labelling (broad slide‐level categories without granular region/feature annotations) further compounds these issues, underscoring the need for standardized curation, prospective annotation and clear case‐selection protocols. Slide‐only models can misinterpret lesions that require multimodal context. In TGCTs, integration of clinical features, imaging and serum tumour markers is often decisive. Mitigations include multi‐site data pooling with stratified external validation, task‐specific endpoints (e.g. LVI detection vs. TIL quantification), abstention/uncertainty policies and explicit performance reporting on rare subtypes. Testis‐specific examples include subtle LVI, focal GCNIS at margins, and heterogeneous mixed‐tumour components, each requiring precise region‐level ground truth to avoid label leakage and biased metrics.

## New Frontiers: Foundation Models and Multimodal Integration in GU Pathology

Pathology increasingly evolves from task‐specific AI algorithms towards foundation models, that is, large‐scale, versatile AI systems adaptable across multiple diagnostic challenges. Interest in multimodal integration, where AI synthesizes data across histology, radiology and genomics, rapidly expands. These advances promise transformative impacts by bridging traditionally isolated clinical domains. Here, we discuss capabilities, current limitations and clinical implications of foundation and multimodal models in GU pathology.

### Breaking Data Silos: Multimodal AI for Unified Diagnostics and Prognostication

Multimodal AI integrates histopathologic findings with radiologic, genomic and clinical data, addressing the inherently multidisciplinary nature of GU cancer evaluation. In prostate cancer, diagnosis traditionally involves independent assessments of biopsy histology, MRI and genomic risk profiles. AI‐driven multimodal integration provides a comprehensive diagnostic and prognostic framework, as early research demonstrates improved prediction of recurrence risk or therapeutic response when combining digitized histology, MRI and genomic markers (e.g. improved AUC from ~0.65 with imaging alone to ~0.70 with multimodal integration).^152^, ^153^

In bladder cancer, integrating cystoscopic images with microscopic histopathology could offer real‐time, comprehensive assessments, predicting tumour grade, invasiveness, and molecular features like FGFR3 mutations, aiding intraoperative decision‐making.^154^, ^155^

The true value of multimodal AI lies in detecting patterns and risks missed by single‐modality analyses. Renal cysts appearing benign on imaging might be better stratified for malignancy when analysed with biopsy histology and clinical data.^156^, ^157^ Likewise, subtle histologic prostate biopsy findings, contextualized with MRI risk scores, may alter diagnostic thresholds.^158^ The systematically, clinically integrative nature of multimodal AI may be used to automatically alert pathologists to discrepancies between imaging and histologic findings, prompting deeper review and potentially reducing diagnostic errors.^159^ By 2030, AI‐generated multimodal diagnostic summaries might routinely combine histologic grade, imaging features and genomic findings into unified risk assessments (e.g. ‘Prostate adenocarcinoma, Grade Group 2, PI‐RADS 5 lesion, TMPRSS2‐ERG fusion positive, intermediate aggregated recurrence risk’).^160^ Comprehensive reports would enhance clinical judgement, supporting nuanced patient management decisions.^161^

Practical integration challenges remain, including interpretive confusion from multiple AI assessments, data weighting concerns and propagation of single‐modality errors. Multimodal AI is best initially implemented within multidisciplinary contexts like tumour boards, where clinicians collaboratively interpret integrated outputs. Procedural changes, such as joint pathology–radiology reviews facilitated by AI summaries, will be necessary for optimal adoption.

### Generalist Whole‐Slide Models: From Narrow AI to Foundation AI

Pathology AI models are traditionally task‐specific, optimized for individual diagnostic tasks or organ systems. Comprehensive pretraining enhances model generalizability, even in less represented areas like renal and testicular pathology. Championing this principle are foundation models, that is, general‐purpose AI systems trained extensively across multiple diseases and tissues. ‘GigaPath’, trained on 1.3 billion image tiles from over 171,000 WSIs across diverse tissues (including GU organs), achieved state‐of‐the‐art performance across various diagnostic benchmarks without explicit task‐specific fine‐tuning.^162^, ^163^

Other generalist models, such as UNI and Microsoft's multimodal BioMedCLIP, demonstrate comparable versatility. BioMedCLIP, trained on millions of biomedical images paired with descriptive captions, matched dedicated pathology models' performance, highlighting how scale and dataset diversity offset narrower, domain‐specific approaches.^164^, ^165^

Foundation models offer practical advantages for GU pathologists, including increased robustness to variations in staining protocols or scanner technologies, reducing frequent revalidation needs.^166^ These models enable niche AI tool development for rare tumour classifications (e.g. oncocytic renal neoplasms), leveraging fine‐tuning on smaller data sets—a democratization analogous to advances seen with large language models.^164^

Foundation model limitations have been obscured by high benchmark performances. Shortcomings are revealed under targeted scrutiny, such as poorer accuracy on rare cancer types or important non‐neoplastic pathology (e.g. placenta) without focused fine‐tuning.^167^ Interpretability is often compromised, as generalist models typically operate as ‘black boxes’ (internal decision process cannot be visualized directly by pathologists).^162^, ^168^, ^169^, ^170^

**Figure 4:**
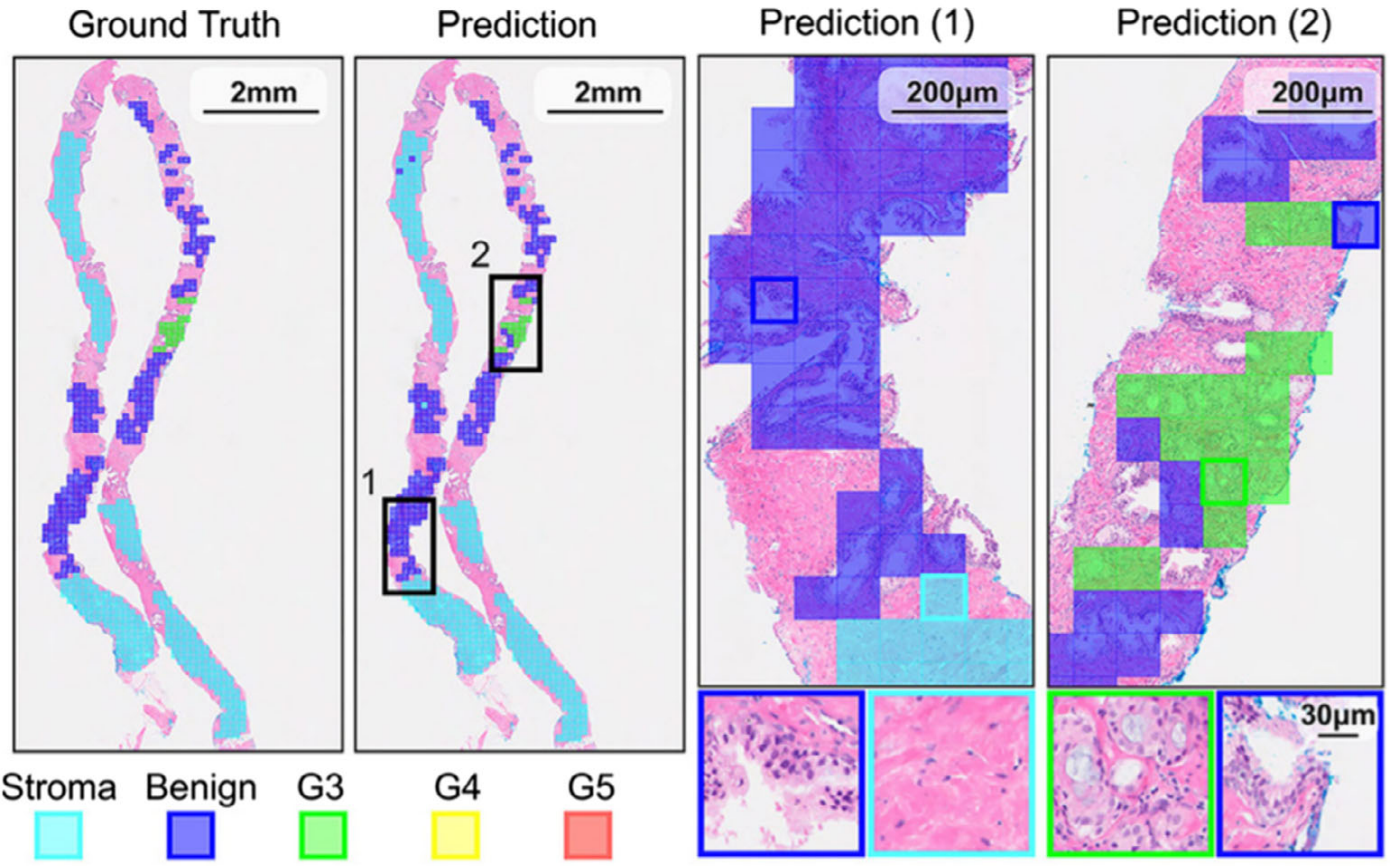
UNI (general‐purpose self‐supervised model) applied to region of interest (ROI) classification of prostate adenocarcinoma.^165^ Whole slide image ground‐truth ROI labels and UNI‐predicted patch labels. Pretrained weights for UNI are publicly hosted on Hugging Face under a research licence, enabling anyone to download and use them. Users can freeze the vision backbone and fine‐tune lightweight heads (e.g. linear probes, attention‐MIL aggregates) on their own labelled slides, adapting UNI's generic histology representations to local tissue prep, scanner settings and diagnostic endpoints. Because the core model is released, academic pathology labs can run all training and inference on‐premises with their own GPUs, meeting privacy regulations while customizing the system for their patient population.^165^

Foundation models in GU pathology will likely thrive within collaborative frameworks. Public release of pretrained model weights by institutions and companies facilitates localized fine‐tuning, allowing pathology departments to tailor AI specifically to their patient populations and clinical scenarios (Figure 4).

Local adaptations integrate the breadth of foundational knowledge with specialized, patient‐specific expertise, effectively blending comprehensive generalist capabilities with precise, targeted diagnostic refinement.

The ‘Virchow’ foundation model further supports this trajectory. Trained using a self‐supervised approach on >1.5 million H&E WSIs, Virchow enabled a pancancer detector that approached clinical product performance overall and outperformed specialist models on several rare histologic variants while using fewer tissue‐specific labels.^171^ The same embeddings boosted biomarker prediction and remained robust to external institution slides, hinting that large in‐domain pretraining can reduce local revalidation needs. As a black box model, Virchow's mainstream deployment is also limited by regulatory uncertainty and persistent gaps for non‐neoplastic pathology.

#### ‘Explainable (interpretable) AI’ (xAI) in pathology

For clinical interpretability, two aspects are paramount: (a) whether the model inherently yields decision‐relevant region information (e.g. attention mechanisms, patch‐level outputs) or requires post‐hoc methods (e.g. Grad‐CAM, occlusion sensitivity), and (b) how clearly the tool displays that information to the user (e.g. region heatmaps, cell‐level counts, confidence/abstention cues). Model scale (small vs. ‘foundation’) and task breadth are orthogonal to these needs. In GU pathology, explainability is operationalized as region‐ and feature‐level evidence that lets pathologists verify focus on clinically relevant patterns (e.g. malignant glands, cribriform/IDC, LVI), detect site/batch‐related shortcuts, and connect model accuracy to safety, auditability and adoption. Table 2 summarizes six ways xAI ties AI to GU practice (Table 2).

**Table 2:** Six xAI functions that tie AI outputs to GU pathology practice^172^, ^173^, ^174^, ^175^

xAI function (tie to practice)	Practical mechanism	GU‐specific examples	User‐facing evidence/artefacts
Region‐level grounding of decisions	MIL/attention, patch‐ranking, saliency/Grad‐CAM when needed	Malignant glands, cribriform/IDC‐P foci, CIS, subtle LVI	Mini‐map with top‐N patches, heatmap overlays, click‐through to WSI context
Feature‐level quantification	Human‐interpretable features (HIFs), object detection and counting	Tumour%, gland counts/size, TIL density, mitotic counts, GCNIS extent	Tabulated counts with CIs, calibration plots, side‐by‐side before/after
Uncertainty and abstention for safety	Confidence calibration, conformal prediction, OOD detectors	Out‐of‐distribution stains/scanners, tiny tissue area; focus/artefact	Case‐level abstain flag; reason codes (e.g. OOD, low tissue); pass‐rate dashboard
Bias/shortcut detection and site effects	Site‐stratified performance, attention sanity checks, counterfactuals	Scanner brand watermark, batch/stain signatures, coverslip edges	Attention not concentrated on non‐tissue regions, site‐wise metrics in reports
Regulatory traceability and clinical auditability	Evidence logging, version control, provenance tracking	Prostate grading calls, negative‐case triage in bladder	Persist heatmaps, patch lists, counts, thresholds, model + data versions with each case
Foundation‐model era: same xAI needs	Prototype retrieval, concept activation, attention in VLMs	Retrieve similar GU patterns; concept ‘cribriform’ scorecards	Prototype gallery with similarity scores, concept‐level rationales

CI, confidence interval; GCNIS, germ cell neoplasia in situ; GU, genitourinary; HIFs, human‐interpretable features; IDC‐P, intraductal carcinoma of the prostate; LVI, lymphovascular invasion; MIL, multiple‐instance learning; OOD, out‐of‐distribution; QMS, quality management system; TILs, tumour‐infiltrating lymphocytes; VLMs, vision–language models; WSI, whole‐slide image.

### Vision‐Language Models: Capturing Pathology's Language and Rare Knowledge

Recent advances in vision–language models build on training with extensive data sets of paired histologic images and descriptive texts, allowing these systems to learn associations between microscopic features and the diagnostic lexicon used by pathologists (e.g. terms such as ‘cribriform’, ‘hobnail’ or ‘Schiller–Duval bodies’). CONCH (CONtrastive Learning from Captions in Histopathology), trained on more than 1.17 million image–caption pairs drawn from authoritative pathology sources, has demonstrated the capacity to generate pathology‐specific descriptions directly from visual input.^176^

Given GU pathology's numerous rare entities, vision‐language models offer significant practical value. Rare tumours, such as uncommon testicular cancers, often lack extensive visual examples but are richly described in pathology literature. Models trained on these descriptive captions leverage textual knowledge to accurately identify these entities in limited‐data scenarios. CONCH's robust performance, including accurate zero‐shot tasks like prostate Gleason grading, highlights the potential for interactive AI systems capable of providing diagnostic suggestions and clear rationales.

Practically, these models can serve as valuable educational and reference tools. Trainees could query descriptive terms such as ‘glomeruloid structures in prostate adenocarcinoma’ and retrieve illustrative images, enhancing diagnostic training. Experienced pathologists could use these models for rapid morphological searches, creating a valuable internal AI‐supported consultative service for unusual or challenging presentations.

A notable challenge specific to vision‐language models is their susceptibility to plausible but incorrect outputs (‘hallucinations’), such as inaccurately reporting lymphovascular invasion or referencing non‐existent studies. These risks necessitate expert oversight and verification, ideally supplemented by retrieval‐based systems to ensure accuracy. While vision‐language models excel at descriptive tasks, they may lack precision in quantitative analyses (e.g. mitotic counting) unless specifically fine‐tuned. Their extensive computational demands may initially restrict clinical deployment to well‐resourced institutions or cloud‐based platforms, raising additional privacy considerations.

Emerging solutions addressing these challenges include interactive pathology assistants such as PathChat: a specialized model combining vision encoders with language models fine‐tuned explicitly for diagnostic pathology queries. Early evaluations demonstrate PathChat's effectiveness in answering diagnostic questions, recommending appropriate ancillary tests and refining differential diagnoses, surpassing more generalized AI models.^177^ Such suggests a future where pathologists routinely engage conversationally with a foundational ‘chatbot’ AI model during case sign‐outs to achieve greater diagnostic accuracy and efficiency.

Establishing clinical trust in vision‐language systems requires increased transparency, interpretability and rigorous validation. Pathologists need clear, evidence‐based visual explanations for AI‐generated recommendations (e.g. diagnostic heatmaps highlighting influential regions). Regulatory approval remains a critical hurdle, necessitating comprehensive clinical trials to demonstrate safety and efficacy before widespread adoption.

Vision‐language models introduce important cultural considerations for clinical practice. Ideally, these AI tools will operate symbiotically with pathologists, democratizing expert diagnostic guidance and helping reduce disparities across diverse clinical environments. Successful integration of vision‐language and multimodal models as trusted clinical tools depends on rigorous validation, controlled deployment and ongoing clinician‐guided refinements.

## Building an AI‐Integrated GU Pathology Practice: A Strategic Roadmap

By the end of this decade, AI will become deeply embedded within GU pathology workflows. This section provides a structured, pragmatic blueprint illustrating how AI could realistically reshape GU pathology by 2030, emphasizing economic viability, governance frameworks, equity considerations and role adaptation within pathology practices.

Successful integration requires strategic planning, rigorous validation and thoughtful evolution of existing roles, procedures and challenges to the deployment of AI tools for routine diagnostics (Table 3).

**Table 3:** Challenges of deployment of AI‐powered diagnostic tools in genitourinary pathology

Category	Barrier
Absence of	Diagnostic concordance: no model achieves perfect accuracy; residual errors can be clinically meaningful*
	Randomized or controlled trials demonstrating the effect of AI assistance on patient outcomes or long‐term clinical utility
	Adequately powered multi‐institutional data sets with external validation and prospective cohorts*
	Established reimbursement pathways for AI‐enabled diagnostics, limiting economic incentives for adoption
Burden of	High capital and operational expenditure required to digitize slides and integrate AI solutions into existing laboratory workflows
Uncertainty of	Model performance can drift when input data (scanner, staining protocol, patient mix) differ from training conditions
	Regulatory pathways for pathology AI devices: heterogeneous and still evolving
	Medico‐legal responsibility for AI‐related misdiagnoses or omissions remains undefined.

* As used in this review, ‘adequately powered’ means sample sizes are pre‐specified by power analysis for the primary endpoint (targeting CI half‐widths ≈±3%–5% at 95% confidence), with external validation across multiple institutions (typically ≥3); for assistive tools, a multi‐reader, multi‐case (MRMC) design (or equivalent) powers reader‐in‐the‐loop effects. Depending on prevalence, this often implies hundreds of disease‐positive cases, with total cases commonly in the low thousands. As an illustration (not a standard): to estimate sensitivity ≈0.90 with ±3% precision at 95% confidence requires ≈385 disease‐positive cases; at 20% prevalence, ≈1925 total cases. Multi‐site sampling (≥3 institutions) is advisable (derivation based on binomial precision formulas for diagnostic accuracy, TRIPOD + AI guidance on a priori sample‐size justification, CAP validation resources and FDA recommendations for multi‐site external validation and MRMC reader studies; Paige Prostate De Novo provides an MRMC precedent with 16 pathologists and 610 WSIs).^178^, ^179^, ^180^, ^181^, ^182^

### Envisioning GU Pathology in 2030: Pathologists as AI‐Enabled Diagnostic Orchestrators

By 2030, GU pathology practices will integrate AI deeply into diagnostic workflows. Upon specimen digitization, AI will automatically conduct preliminary analyses. For prostate biopsies, AI systems will rapidly triage benign cores from suspicious ones, annotating areas of concern and providing preliminary findings such as tumour length, Grade Group or perineural invasion. Pathologists will efficiently verify AI annotations, directing attention primarily towards ambiguous or subtle findings. Continuous AI learning will integrate pathologist corrections, progressively refining diagnostic accuracy.

Renal pathology AI systems will quickly assess partial nephrectomy specimens, confirm clear margins, classify tumours and correlate histology with imaging findings. Pathologists, freed from repetitive quantification tasks, will focus more on higher‐level diagnostics, radiologic–pathologic correlations and multidisciplinary collaboration.

Bladder cancer intraoperative consultations will benefit significantly, with AI rapidly analysing frozen sections and immediately communicating margin issues directly to surgical teams, enhancing real‐time clinical decisions.

Operationally, AI‐powered dashboards will continuously track diagnostic quality metrics, flagging deviations like unusual ancillary test ordering patterns, enabling proactive quality assurance and targeted retraining.

### Economic Considerations and Return on Investment

The economic rationale for adopting AI varies across institutional contexts and should be assessed alongside digital pathology (WSI) business cases and local constraints (scanner/IMS/LIS, storage, staffing).

- *Large Academic Centres*: Return on Investment (ROI) can accrue via efficiency and scale, that is, shorter review times, fewer slide movements/retrievals and targeted reduction of ancillary testing when supported by prospective evidence. Prospective and real‐world evaluations of prostate AI report ~22%–30% faster review for malignant and benign cases, supporting higher peak throughput. Digital pathology cost models from large centres report multimillion‐dollar savings over 5 years (e.g. $18M in a large integrated health system, $1.3M at MSK from workflow and archival efficiencies), illustrating the order of magnitude achievable at scale (exact figures are sitedependent).^183^, ^184^ Early implementation and HTA reports also show improved sensitivity/concordance with AI assistance, which is part of the economic case through safety and rework avoidance.^185^
- *Mid‐sized Community Hospitals*: These sites typically favour SaaS/percase commercial models. NICE and HTA documents describe subscription pricing that starts around £1 per slide with a one‐off LIS integration fee (typically ≥£15 k); total cost varies with case volume and storage. Health Technology Wales' evidence appraisal modelled AI‐assisted prostate biopsy review as cost‐effective at an ICER of £13,278/QALY (below the UK threshold), with reduced additional testing and overall time benefits reported in the clinical studies it summarized. Where local volumes are moderate, focusing on high‐yield use cases (e.g. prostate biopsy assistance) and staged rollout improves the business case.^185^
- *Low‐resource and remote settings*: Economic benefit often derives from capacity expansion and avoided transfers rather than direct cash savings. Regional programs have maintained intraoperative services without an onsite pathologist, thereby avoiding patient transfers and delays.^186^ WHO guidance supports digital/teleinterventions to strengthen health systems in underserved areas (infrastructure and governance permitting). Modern studies and guidance on *remote digital reporting* further substantiate feasibility and safety, which are prerequisites for any ROI in these contexts.^187^
- *Private labs and group practices*: Common ROI drivers include shorter review times and higher peak throughput with AI‐assisted triage^188^; targeted IHC utilization (with emerging trial data showing reduced IHC costs while maintaining diagnostic safety)^15^, ^189^; fewer physical shipments/‘sendouts’ due to digital sharing of slides and TAT improvements, a long‐established key performance indicator (KPI) linked to client satisfaction and retention.^190^, ^191^ Casebased software as a service (SaaS) pricing (per slide/per case subscriptions) lets private labs align costs with volume; paired with measured time/IHC savings and cost‐effectiveness models, this can support near cash flow neutrality within renewal cycles in some settings.^38^, ^192^ In the United States, Category III add‐on CPT codes (0751T–0763T; 0827T–0856T) allow reporting of slide digitization performed for primary diagnosis (with or without AI), but payment varies by payer and is not guaranteed. Several Medicare Administrative Contractors currently make no additional payment for these add‐on codes. Direct algorithm‐specific reimbursement is not established.

In all scenarios, budgeting should balance upfront (infrastructure, software, integration, training, validation and QMS) and ongoing costs against concrete benefits (efficiency, error reduction/consistency, avoided shipments, improved TAT and targeted ancillary testing) and HTA‐style cost‐effectiveness where available. Recent European assessments and models (e.g. HTW; Swedish microsimulation) provide practical precedents on cost‐effectiveness and the scale of potential workload reduction in prostate biopsy pathways.

### Workflow Transformation: Strategic Automation and Role Reinvention

Successful AI implementation requires strategic decisions about workflow elements:

- *Automation opportunities*: Highly standardized tasks (e.g. Gleason grading, routine immunohistochemical quantifications, straightforward frozen‐section evaluations) are prime candidates for AI‐driven automation, improving efficiency, accuracy and consistency.
- *Processes requiring reinvention*: Quality assurance may evolve from retrospective audits to real‐time, targeted AI‐driven evaluations. Pathology education and certification will increasingly embed AI competencies. Reports will become structured with AI‐generated data, shifting pathologist roles towards verification, interpretation and contextual integration. AI‐generated summaries will enhance multidisciplinary communication.
- *Human‐centric roles to retain*: Integrated diagnostic reasoning, nuanced clinical scenario management, ethical decisions, regulatory oversight and patient‐centred communication remain firmly under human leadership, maintaining accountability and ethical standards.
- *Emerging capabilities*: AI will introduce novel capabilities, for example, rich, data‐driven diagnostic reports, advanced visualizations and predictive analytics. Workflow orchestration (AI‐driven case prioritization) and predictive diagnostics will augment pathologists' prognostic roles, enhancing personalized treatment recommendations.

Carefully managing these transitions, with pathologists actively involved, ensures AI enriches rather than diminishes professional roles. Delegating routine tasks to AI allows pathologists to engage deeply with complex cases, interdisciplinary collaboration and direct clinical impact, enhancing professional satisfaction and patient care quality.

### Implementation Strategy (‘VALIDATED’ and ‘ORCHESTRATE’)

Implementing AI in pathology demands structured, iterative planning, validation and governance, guided by regulatory bodies (CAP, WHO, FDA), and validated through systematic evaluation and stakeholder engagement.

**Figure 5:**
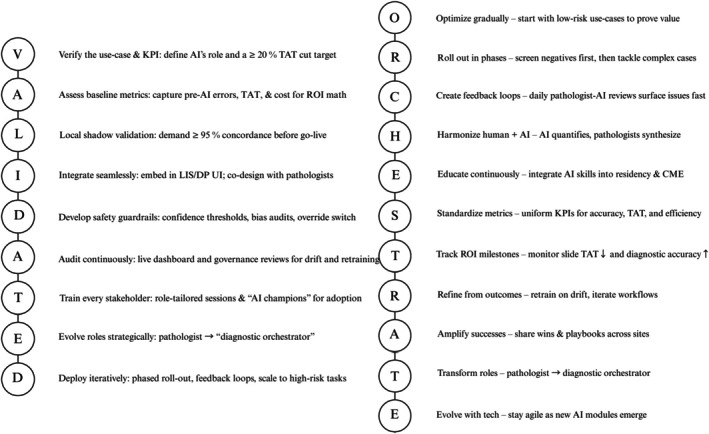
The VALIDATED–ORCHESTRATE blueprint: high‐impact implementation frameworks for AI in GU pathology. ‘Quick‐list’ reference guide depicting ‘VALIDATED’ and ‘ORCHESTRATE’ frameworks for AI implementation in GU pathology. Acronyms are expanded within the figure for readability (see Tables 4 and 5 for checklist detail).

To ensure both technical success and cultural adoption, positioning pathology departments for sustainable AI integration, we propose the VALIDATED framework as a practical approach for AI integration (Table 4). The complementary governance and operations frameworks are summarized in Figure 5 and detailed in Tables 4 and 5.

**Table 4:** The VALIDATED framework for AI implementation in GU pathology

*V—Verify use case and define scope*
Clearly delineate AI's intended role (primary diagnostics, secondary quality control, decision support). Establish measurable success criteria upfront (e.g. reduce slide review time by 20% while maintaining accuracy). Clear initial intent mitigates operational disruptions and enhances utility
*A—Assess baseline performance metrics*
Document current performance metrics including TATs, error rates and resource utilization. These baseline measurements enable quantitative assessment of AI impact and ROI calculations
*L—Local validation in shadow mode*
Perform rigorous, local parallel ‘shadow mode’ validation, comparing AI outputs directly against pathologist interpretations. Aim for ≥95% concordance before operational implementation. Analyse systematic discrepancies, fine‐tune on local data and continuously reassess performance, including rare and challenging cases.
*I—Integrate with existing systems*
Integrate AI seamlessly into LIS and digital pathology platforms, emphasizing user‐friendly interfaces. Engage pathologists directly in interface testing and design to avoid cognitive overload and missed information, ensuring practical adoption
*D—Develop safety guardrails*
Develop clear safety mechanisms—confidence thresholds, abstention guidelines, transparent explanatory outputs (e.g. diagnostic heatmaps)—ensuring AI supports rather than autonomously drives decisions. Establish explicit override protocols, fairness guidelines and bias mitigation strategies.
*A—Audit continuously*
Implement ongoing quality monitoring of AI performance, systematically tracking discordances, false‐positive/negative rates and other systematic issues. An oversight committee should regularly review performance metrics, directing updates, retraining and strategic adjustments, ensuring continued alignment with clinical and regulatory standards
*T—Train all stakeholders*
Comprehensive training for pathologists, technologists and clinicians must clarify AI capabilities, limitations and interpretation nuances. Address anxieties proactively, framing AI as a supportive partner. Identify and empower departmental ‘AI champions’
*E—Evolve roles strategically*
Recognize that AI implementation transforms professional roles. Pathologists evolve from pure diagnosticians to diagnostic orchestrators, integrating diverse data streams. Support this transition through targeted education and gradual responsibility shifts
*D—Deploy with measured confidence*
Phased rollouts, continuous feedback loops and responsive adjustments promote smoother transitions and long‐term sustainability. Begin with lower‐risk applications before expanding to critical diagnostic tasks.

**Table 5:** The ORCHESTRATE framework for AI operations in GU pathology

*O—Optimize workflow gradually*
Begin with lower‐risk applications before expanding to critical diagnostic tasks. This measured approach allows teams to build confidence and refine processes without disrupting essential services
*R—Roll out in phases*
Implement AI capabilities incrementally, starting with screening negative cases before progressing to complex diagnostics. Each phase builds on previous successes
*C—Create feedback loops*
Establish robust communication channels between pathologists, technologists and AI systems. Regular touchpoints ensure the early identification of issues and opportunities for optimization
*H—Harmonize human–AI collaboration*
Design workflows that leverage the strengths of both human expertise and AI efficiency. Pathologists focus on complex integration while AI handles routine quantification
*E—Educate continuously*
Embed AI competencies into daily practice through ongoing training, from residency programs to board certifications
*S—Standardize quality metrics*
Develop consistent measures for AI performance, diagnostic accuracy and workflow efficiency across all implementations
*T—Track ROI milestones*
Monitor key performance indicators aligned with institutional goals, from TAT reductions to diagnostic accuracy improvements
*R—Refine based on outcomes*
Use performance data to continuously improve AI algorithms and workflow integration
*A—Amplify successful practices*
Share wins across departments and institutions to accelerate adoption and learning
*T—Transform roles strategically*
Support the evolution from traditional diagnostician to diagnostic orchestrator through targeted education and gradual responsibility shifts
*E—Evolve with technology*
Remain adaptable as AI capabilities advance, ensuring your practice stays at the forefront of innovation.

### From Foundation to Execution: The ORCHESTRATE Principles

While the VALIDATED framework provides the foundational structure for AI adoption, successful daily implementation requires operational excellence. We present the ORCHESTRATE framework to guide practical execution (Table 5).

VALIDATED and ORCHESTRATE together provide a complete blueprint for AI transformation: VALIDATED ensures safe, systematic implementation, whereas ORCHESTRATE drives daily operational excellence. This dual‐framework approach positions pathology departments.

The frameworks presented here provide reproducible pathways for AI adoption across diverse institutional settings. By following these systematic approaches, pathology departments can navigate the complex transition from traditional to AI‐enhanced practice while maintaining diagnostic excellence.

## The Roadmap and the Destination for Successful AI Integration in GU Pathology

**Figure 6:**
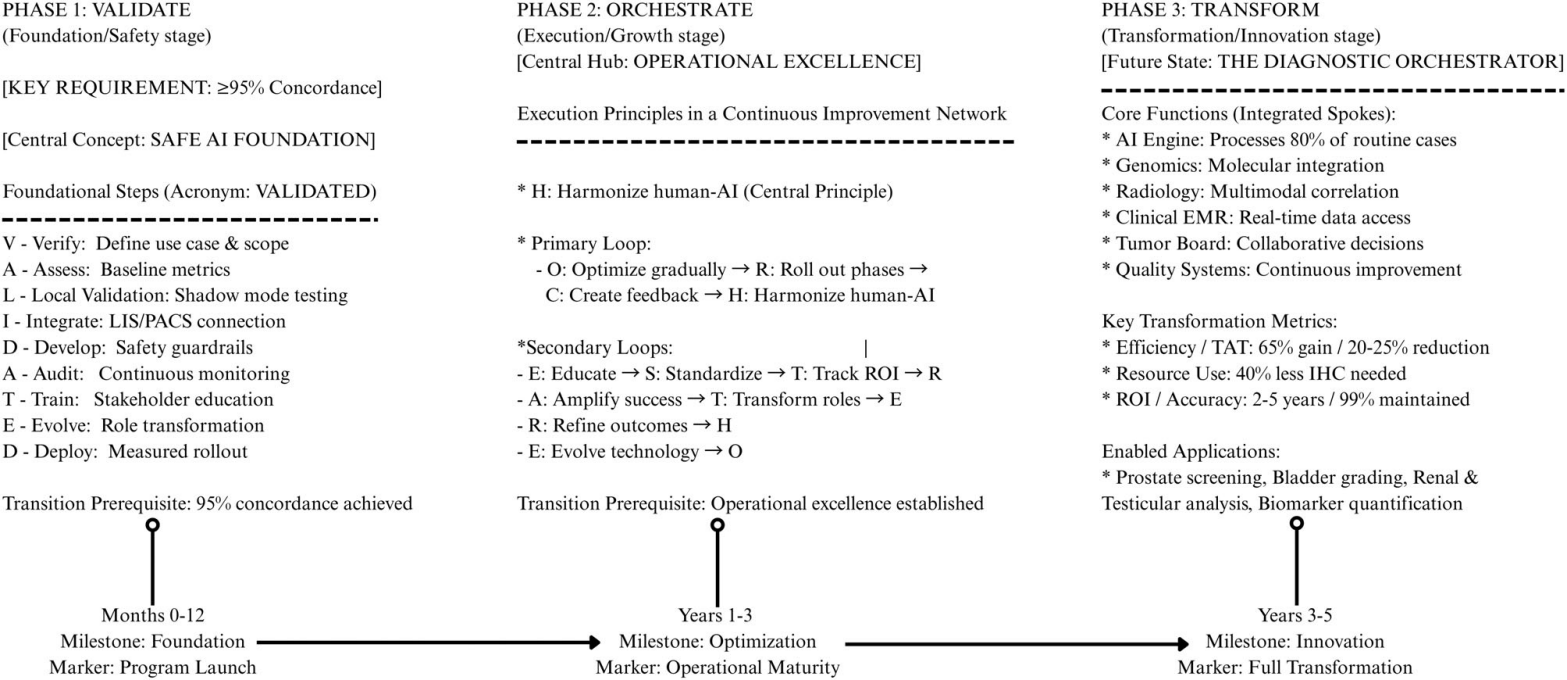
Three‐phase, dual‐framework implementation model: foundational validation to full operational transformation. The Dual‐Framework Implementation Model illustrating the pathway from foundational AI validation through operational excellence to the future state of pathology. The VALIDATED framework (left) establishes safe implementation, ORCHESTRATE principles (centre) drive daily execution, culminating in the Diagnostic Orchestrator Model (right), where pathologists leverage AI to deliver enhanced patient care. Timeline represents typical progression for academic medical centres.

**Figure 7:**
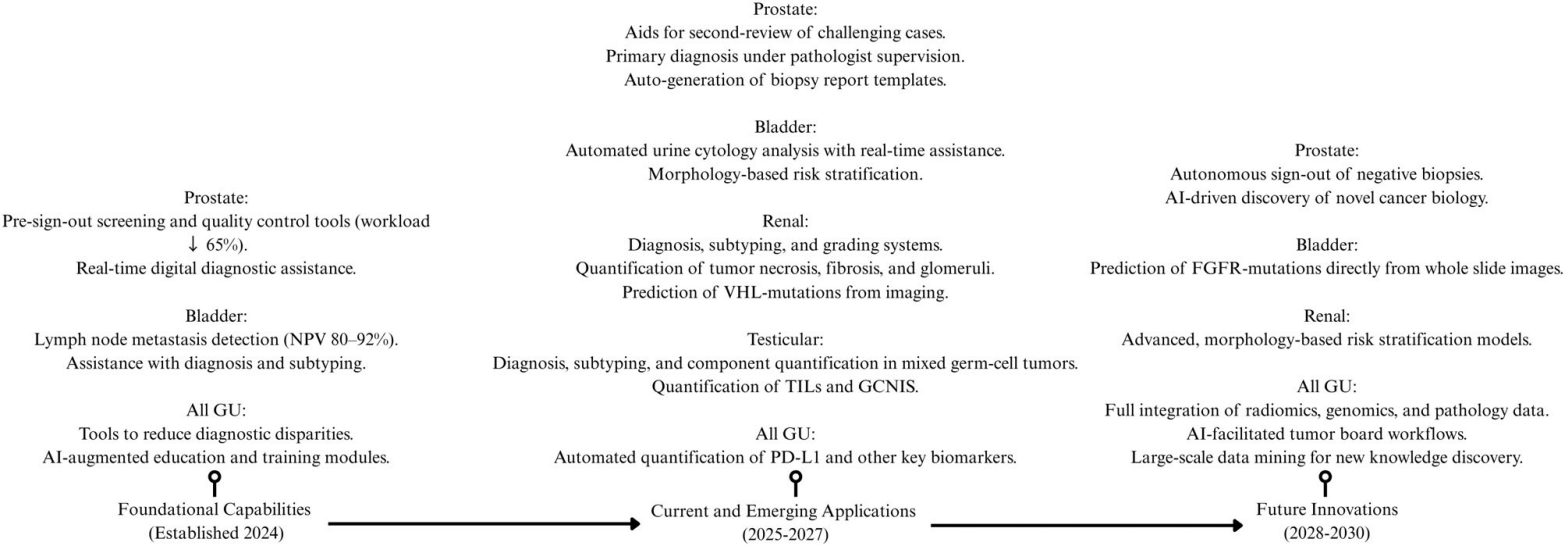
The AI application landscape roadmap for 2024–2030 in GU pathology. Timeline reflects the authors' synthesis of (i) regulatory milestones (e.g. 2021 FDA De Novo clearance), (ii) implementation protocols and pragmatic trials and (iii) microsimulation and real‐world adoption reports cited in this review. Years indicate envisioned adoption inflection points given current regulatory, technical and infrastructure trends.

The pathologist's role will evolve from traditional diagnostician to diagnostic orchestrator—integrating diverse diagnostic data into cohesive clinical narratives. This transformation, illustrated in Figure 6, follows a predictable progression from foundational validation through operational excellence. Figure 7 complements this journey by mapping specific AI applications across the maturity spectrum—from currently operational tools to future innovations enabling autonomous diagnostics. Together, these visualizations provide both the roadmap (how to implement) and the destination (what to implement) for successful AI integration in GU pathology.

## Funding

No specific funding was received for this work. The authors received no financial support from any public, commercial or not‐for‐profit funding agency for the preparation of this manuscript.

## Conflict of interest

The authors declare no conflicts of interest related to the content of this manuscript. Any affiliations or activities that could appear to influence the work have been disclosed to the journal.

## Author contributions

Conceptualization: A.U.P., A.V.P., S.S. Methodology: A.U.P. Investigation and Data curation (literature synthesis): A.U.P., S.S. Visualization: A.U.P. Writing (original draft): A.U.P. Writing (review and editing): A.U.P., S.S., A.V.P. Supervision: S.S., A.V.P. Project administration: S.S., A.V.P. All authors read and approved the final manuscript.

## Data Availability

This article is a narrative review. No new data sets were generated or analysed. All data supporting the statements herein are drawn from previously published studies that are cited in the reference list; any additional materials are available from the corresponding author upon reasonable request.
